# Sulforaphane Causes Epigenetic Repression of *hTERT* Expression in Human Breast Cancer Cell Lines

**DOI:** 10.1371/journal.pone.0011457

**Published:** 2010-07-06

**Authors:** Syed M. Meeran, Shweta N. Patel, Trygve O. Tollefsbol

**Affiliations:** 1 Department of Biology, University of Alabama at Birmingham, Birmingham, Alabama, United States of America; 2 Comprehensive Cancer Center, University of Alabama at Birmingham, Birmingham, Alabama, United States of America; 3 Center for Aging, University of Alabama at Birmingham, Birmingham, Alabama, United States of America; 4 Nutrition Obesity Research Center, University of Alabama at Birmingham, Birmingham, Alabama, United States of America; Oregon State University, United States of America

## Abstract

**Background:**

Sulforaphane (SFN), an isothiocyanate found in cruciferous vegetables, is a common dietary component that has histone deacetylase inhibition activity and exciting potential in cancer prevention. The mechanisms by which SFN imparts its chemopreventive properties are of considerable interest and little is known of its preventive potential for breast cancer.

**Principal Findings:**

We found that SFN significantly inhibits the viability and proliferation of breast cancer cells *in vitro* while it has negligible effects on normal breast cells. Inhibition of telomerase has received considerable attention because of its high expression in cancer cells and extremely low level of expression in normal cells. SFN treatment dose- and time-dependently inhibited human telomerase reverse transcriptase (*hTERT*), the catalytic regulatory subunit of telomerase, in both MCF-7 and MDA-MB-231 human breast cancer cells. DNA methyltransferases (DNMTs), especially DNMT1 and DNMT3a, were also decreased in SFN-treated breast cancer cells suggesting that SFN may repress *hTERT* by impacting epigenetic pathways. Down-regulation of DNMTs in response to SFN induced site-specific CpG demethylation occurring primarily in the first exon of the *hTERT* gene thereby facilitating CTCF binding associated with *hTERT* repression. Chromatin immunoprecipitation (ChIP) analysis of the *hTERT* promoter revealed that SFN increased the level of active chromatin markers acetyl-H3, acetyl-H3K9 and acetyl-H4, whereas the trimethyl-H3K9 and trimethyl-H3K27 inactive chromatin markers were decreased in a dose-dependent manner. SFN-induced hyperacetylation facilitated the binding of many *hTERT* repressor proteins such as MAD1 and CTCF to the *hTERT* regulatory region. Depletion of CTCF using siRNA reduced the SFN-induced down-regulation of *hTERT* mRNA transcription in these breast cancer cells. In addition, down-regulation of *hTERT* expression facilitated the induction of cellular apoptosis in human breast cancer cells.

**Significance:**

Collectively, our results provide novel insights into SFN-mediated epigenetic down-regulation of telomerase in breast cancer prevention and may open new avenues for approaches to SFN-mediated cancer prevention.

## Introduction

Epidemiological studies have consistently shown that an increased dietary intake of fruits and vegetables is strongly associated with reduced risk of developing chronic diseases, such as cardiovascular disease, diabetes, and cancer [1]–[2]. Sulforaphane (SFN), an isothiocyanate naturally rich in widely consumed cruciferous vegetables such as broccoli, broccoli sprouts, cabbage and kale, has been shown to reduce the risk of developing many common cancers, including breast cancer [3]–[7]. SFN was first identified as a potent inducer of phase 2 detoxification enzymes [8], and studies have also found other anti-carcinogenic as well as anti-oxidant mechanisms including induction of caspases, induction of glutathione S-transferase, inhibition of cytochrome P450 isoenzymes and reduction of the DNA binding ability of nuclear factor-κB [6]–[8]. However, there has been growing interest in epigenetic regulation by SFN in chemoprevention due to its histone deacetylase (HDAC) inhibition activity [9]–[12]. The HDAC inhibition activity of SFN has been shown to lead to an increase in the global and local histone acetylation status of a number of genes [9], [13]–[14]. SFN-mediated epigenetic alterations are believed to be strongly involved in the process of cancer chemoprevention by altering the expression of various genes, including tumor suppressor genes in various cancers [5].

The human telomerase reverse transcriptase (*hTERT*) gene that encodes the catalytic subunit of telomerase is a highly epigenetically-regulated gene, and is widely expressed in more than 90% of human cancers but not in normal somatic cells. *hTERT* is a promising target for cancer therapeutics and an important marker for the diagnosis of malignancy [15]–[16]. This critical gene is regulated by several epigenetic alterations at promoter sites including histone acetylation and promoter methylation [15]–[17]. Histone acetylation and deacetylation are dynamic processes typically regulated by histone acetyltransferases (HATs) and HDACs, respectively. HDAC inhibitors enable HAT co-activator complexes to transfer acetyl groups to lysine residues in histones. This leads to an open chromatin structure which facilitates the binding of various transcription factors such as c-MYC, MAD1 and CTCF to gene promoters for the activation or repression of genes, including *hTERT* [17]–[19]. In addition to histone acetylation as a form of epigenetic control of *hTERT* expression, promoter DNA methylation and histone methylation also play significant roles in *hTERT* regulation [19]–[20]. Convincingly, the *hTERT* promoter region is embedded in a CpG island (positions −1100 to +1500), and this region is mostly hypermethylated by specific DNA methyltransferases (DNMTs) in cancer cells except a short region in the *hTERT* core promoter (positions −279 to +5) [21]. The aberrant methylation pattern in the *hTERT* 5′-regulatory region prevents the binding of the methylation-sensitive CTCF repressor to the first exon of *hTERT* [22]. *hTERT* regulatory region hypermethylation has been associated with increased *hTERT* expression, whereas demethylation of this region inhibits *hTERT* transcription [21]–[22]. This phenomenon is opposite to the general model of gene activation, in which the presence of methylated cytosines in a promoter typically inhibits gene transcription.

In addition to histone acetylation and promoter methylation, histone methylation- mediated transcriptional regulation of *hTERT* expression has emerged. Histone acetylation-mediated transcriptional binding of MAD1 recruits RBP2 (a histone demethylase) to the *hTERT* promoter and reduced *hTERT* mRNA expression is accompanied by H3 lysine-4 demethylation [23]. Studies have shown that the HDAC inhibitor, trichostatin A (TSA), induces hyperacetylation of histones at the *hTERT* proximal promoter and directly transactivates the *hTERT* gene in normal human-telomerase negative cells [24]–[25]. In contrast, many studies have also shown that HDAC inhibitors suppress *hTERT* expression in various cancer cells including prostate, leukemic and oral squamous cell carcinoma [18], [26]–[27]. Reports on telomerase inhibition by HDAC inhibitors are controversial, however, most studies have focused on down-stream mechanisms of *hTERT* inhibition such as apoptosis and cell cycle arrest rather than how the HDAC inhibitors regulate *hTERT* expression. Therefore the present study was undertaken to evaluate the complete epigenetic regulation of *hTERT* expression and its promoter alterations in the apoptosis process induced by SFN in human breast cancer cells. Our results indicate that SFN-induced histone acetylation allows transcriptional repressors to bind to the *hTERT* 5′-regulatory region. Further, SFN-mediated demethylation of CpG sites in the exon 1 region of the *hTERT* gene via down-regulation of DNMTs and induction of RBP2, allows the CTCF repressor of *hTERT* to bind to exon 1 of the *hTERT* gene thereby contributing to the inhibition of *hTERT* expression. These findings reveal for the first time that SFN alters the methylation status of the *hTERT* regulatory region at CpG sites on exon 1 but not in the promoter region. Collectively, our studies indicate that changes in the histone modifications of the *hTERT* promoter and DNA demethylation of *hTERT* exon 1 lead to inhibition of cellular growth and the induction of apoptosis of human breast cancer cells in response to the SFN chemoprevention compound.

## Materials and Methods

### Cell culture and cell growth assay

All human cell lines were obtained from the American Type Culture Collection (ATCC, Manassas, VA). Breast cancer MCF-7 and MDA-MB-231 cells were grown in Dulbecco's modified Eagle's medium (DMEM) (Mediatech Inc, Manassas, VA) supplemented with 10% fetal bovine serum (Atlanta Biologicals, Lawrenceville, GA) and 1% penicillin/streptomycin (Mediatech). Normal control MCF10A cells were obtained from ATCC and maintained in DMEM/F-12 medium (Mediatech) supplemented with 5% equine serum (Atlanta Biologicals), 10 µg/ml of human insulin (Sigma, St. Louis, MO), 20 ng/ml of epidermal growth factor (Sigma), 100 ng/ml of cholera endotoxin (Sigma), 0.5 µg/ml of hydrocortisone (Sigma), 2 mM L-glutamine and 1% penicillin/streptomycin (Mediatech). MCF10A is a non-tumorigenic human breast epithelial cell line originally isolated from a 36-year-old Caucasian female. It has been frequently used as a normal human breast cell control [28]–[30]. R,S-sulforaphane (LKT Laboratories, Minneapolis, MN) was prepared in DMSO and stored at a stock concentration of 10 mmol/L at −20°C. Twenty-four hours after seeding the cells, SFN was added to the culture medium at indicated concentrations and the maximum concentration of DMSO was 0.1% (v/v) in the medium. Cells treated only with DMSO served as a vehicle control. For cell growth assay, total viable cell numbers were calculated using a hemocytometer and plotted against number of treatment days. Cells were washed and treated with fresh SFN every three days of culture.

### Colonogenic assay

Approximately 500 cells were seeded into six-well plates in triplicate for each group and allowed to adhere overnight. Thereafter, cell culture medium was changed and cells were treated with 0, 5, 10, 15 and 20 µmol/L SFN. The cells were allowed to incubate at 37°C in the incubator undisturbed for 15 days. During this period each individual surviving cell would proliferate and form colonies. On day 15, the colonies were washed with cold phosphate buffer saline, fixed with cold 70% ethanol and stained with 0.25% trypan blue solution. The colonies that had ≥50 cells/colony were counted and expressed as percent control.

### Total RNA extraction, RT-PCR, and real-time quantitative PCR

Total cellular RNA was isolated from cultured cells using an RNeasy mini kit (Qiagen, Valencia, CA) according to the manufacturer's instructions. Two micrograms of total RNA was reverse-transcribed into cDNA using the iScript cDNA synthesis kit (Bio-rad, Hercules, CA). The PCR primer sets were follows: sense5′-CGG AAG AGT GTC TGG AGC AA-3′ and anti-sense 5′-GGA TGA AGC GGA GTC TGG A-3′, at Tm 52°C for *hTERT*; 5′-TTA CAC GTG TCC ACG GCG TTC-3′ and anti-sense 5′-GCT TGT ATG TGT CCC TGC TGG CA-3′, at Tm 59°C for CTCF; sense 5′-ACC ACA GTC CAT GCC ATC AC-3′ and anti-sense 5′- TCC ACC CTG TTG CTG TA-3′, at Tm 54°C for *GAPDH*. The reaction conditions were 35 cycles of 94°C for 30 sec, Tm°C for 30 sec and 72°C for 25 sec. *GAPDH* was used as an internal loading control. Real-time quantitative PCR was carried out in a Bio-Rad MyiQ thermocycler (Bio-rad, Hercules, CA) using Platinum SYBR Green detection system (Invitrogen, Carlsbad, CA). The primer sets were follows: sense 5′-AGG GGC AAG TCC TAC GTC CAG T-3′ and anti-sense 5′-CAC CAA CAA GAA ATC ATC CAC C-3′ for *hTERT*, and sense5′- GAA GGT CGG AGT CAA CGG ATT T-3′ and anti-sense 5′- ATG GGT GGA ATC ATA TTG GAA C-3′ for *GAPDH*. Both primers have a Tm of 60°C. The calculations for determining the relative level of gene expression were made using the cycle threshold (*C*_t_) method. The mean *C*_t_ values from duplicate measurements were used to calculate the expression of the target gene using the formula: fold change in gene expression, 2^−ΔΔCt^ = 2^−{ΔCt (SFN-treated samples)-^ ^ΔC*t* (untreated control)}^, where ΔC*_t_* = C*_t_* (hTERT)- C*_t_* (GAPDH).

### Telomerase activity assay

Telomerase activity was measured using TeloTAGGG telomerase PCR ELISA kit (Roche applied science, Indianapolis, IN) according to the manufacturer's protocol. Three micrograms of protein from total cell lysates was added to the reaction mixture, and the generated telomere product was PCR amplified using 30 cycles (25°C for 20 min, 94°C for 5 min, 94°C for 30 sec, 50°C for 30 sec, 72°C for 90 sec and 72°C for 10 min). PCR amplified products (5 µL) were used for ELISA assays, and the level of telomerase activity was expressed as an arbitrary unit of absorbance at OD_450_–OD_690_.

### Western blot analysis

Protein was extracted from cultured cells using the RIPA lysis buffer (Upstate Biotechnology, Lake Placid, NY) following the manufacturer's directions. Equal amounts of protein were resolved on a 10% SDS-polyacrylamide gel and transferred onto nitrocellulose membranes. After incubation in blocking buffer for 1 h, the membranes were incubated with the primary antibodies specific for DNMT1, DNMT3a, DNMT3b, CTCF, RBP2 (Santa Cruz Biotechnology, Santa Cruz, CA) and β-actin (Cell Signaling, Danvers, MA). The blot was then washed with Tris-Buffered Saline (TBS) with 0.05% (v/v) Tween-20 and incubated with specific secondary antibody conjugated with horseradish peroxidase. Protein bands were then visualized using the ECL detection system (Santa Cruz Biotechnology) following the protocol of the manufacturer. The bands were analyzed by using Kodak 1D 3.6.1 image software for the intensity and normalized with respective β-actin. The mean values for the control group (nontreated) were assigned the value 1 (arbitrary unit), and comparison was then made with densitometry values of other SFN treatment groups.

### HDAC activity assay

Nuclear extract (20 µg) from the SFN-treated as well as untreated cells were assayed for HDAC activity using a colorimetric HDAC assay kit (Active Motif, Carlsbad, CA) according to the manufacturer's instruction. Once the acetylated substrate, BOC-(Ac)Lys-pNi-troanilide, is deacetylated, a colored product results that absorbs maximally at 405 nm. No enzyme control and TSA-positive controls were included and all reactions were setup in triplicate.

### HAT activity assay

HAT activity was determined using the colorimetric HAT activity assay kit (Epigentek, Brooklyn, NY) according to the manufacturer's protocol. Nuclear extract from the SFN-treated as well as untreated cells were assayed for HAT activity. The reaction was initiated by adding 20 µg of nuclear extracts, containing active HATs, to the active-histone coated ELISA plate and incubated for 60 min at 37°C. Acetylated histones were captured by specific antibodies and followed by detection antibodies tagged with color compound. The enzymatic color development was directly proportional to HAT activity measured at 450 nm.

### DNMT activity assay

Nuclear extract from the SFN-treated as well as untreated cells were assayed for DNMT activity using a colorimetric DNMT activity assay kit (Epigentek, Brooklyn, NY) according to the manufacturer's instruction. The reaction was initiated by adding 20 µg of nuclear extracts, containing active DNMTs, to the unique cytosine-rich DNA substrate coated ELISA plate and incubated for 60 min at 37°C. The methylated DNA can be recognized with anti-5-methylcytosine antibody. The amount of methylated DNA, which is proportional to enzyme activity, is calorimetrically quantified at 450 nm.

### Bisulfite sequencing analysis

To assess the methylation status of the *hTERT* promoter, sodium bisulfite methylation sequencing was performed as described previously [31]. Approximately 1 µg of genomic DNA was used for bisulfite modification using the EpiTect-Bisulfite modification kit following the manufacture's protocol (Qiagen, Valencia, CA). Modified DNAs were then amplified by PCR using Go Taq mix (Promega, Madison, WI). Primers and PCR-conditions were followed as described by Choi *et al* [19]. Following PCR amplification, purified bands were cloned using a pGEM-T cloning kit (Promega). Plasmid DNA was isolated using QIAprep Spin Miniprep kit (Qiagen). Plasmid DNAs were sequenced using the 3730 DNA Sequencer (Applied Biosystems, Foster City, CA).

### Chromatin immunoprecipitation analysis

Chromatin immunprecipitation (ChIP) analysis was performed using the EZ-ChIP kit (Cat#17-371; Lot# DAM1556786; Upstate Biotechnology) according to the manufacturer's instructions. The antibodies used in the ChIP assays were ChIP-validated acetyl-histone H3 (Cat#06-599; Lot#DAM1422332; Upstate Biotechnology), acetyl-histone H3K9 (Cat#07-352; Lot#DAM1394804; Upstate Biotechnology), acetyl-histone H4 (Cat#06-598; Lot#31991; Upstate Biotechnology), trimethyl-histone H3K27 (Cat#07-449; Lot#DAM1421462; Upstate Biotechnology), trimethyl-histone-H3K9 (Cat#07-442; Lot#DAM1411287; Upstate Biotechnology), MAD1 (Cat#05-1500; Lot#NG1578247; Upstate Biotechnology), c-MYC (Cat#06-340; Lot#22590; Upstate Biotechnology), HDAC1 (Cat#SC-8410; Lot#D2706; Santa Cruz Biotechnology) and CTCF (Cat#SC-28198; Lot#A2508; Santa Cruz Biotechnology). No antibody control was also used to check ChIP efficiency. ChIP-purified DNA was quantified by using quantitative-PCR (qPCR) on using Platinum SYBR Green detection system (Invitrogen, Carlsbad, CA) as described by Anderson *et al* [32]. Briefly, extracted DNA from each immunoprecipitation was resuspended in 10 µL nuclease free water. In parallel, the input DNA stored as 10% of total lysate was resuspended in 100 µL nuclease free water. Real-time PCR was performed in 25 µL volumes by using Platinum SYBR Green detection system (Invitrogen). The primers for the *hTERT* promoters were forward-5′-TCC CCT TCA CGT CCG GCA TT-3′, reverse-5′-AGC GGA GAG AGG TCG AAT CG-3′.

### Small interfering RNA (siRNA) knock-down of CTCF

Approximately 3×10^5^ cells per well was placed in a 6-well plate and allowed to incubate overnight. The CTCF siRNA (Santa Cruz Biotechnology) was made into 10 µM stock using nuclease free water and 9 nM siRNA was delivered to the cells using the Silencer siRNA Transfection kit (Ambion/Applied Biosystems, TX, USA) according to the manufacturer's instructions. siCONTROL Non-Targeting siRNA (Santa Cruz Biotechnology) was used as a negative control. Cells were harvested and checked for CTCF knock-down after 3 and 6 day intervals using western blot analysis. SFN-treated and non-treated cells were used to harvest RNA for PCR reactions using total RNA extraction and RT-PCR procedures described in previous sections.

### Apoptosis assay

Breast cancer cells transfected with CTCF and control siRNA as well non-transfected cells were treated with 10 µM SFN for 6 days. The cells were then lysed with nuclei lysis buffer provided for apoptosis assays using the Cell Death Detection ELISA Kit (Roche, Palo Alto, CA) as described previously [33]. Briefly, the cytoplasmic histone/DNA fragments were extracted from SFN-treated and untreated cells and incubated in microtiter plate modules coated with antihistone antibody. Subsequently, the peroxidase-conjugated anti-DNA antibody was used for the detection of immobilized histone/DNA fragments, followed by color development with 2,2′-azinobis(3-ethylbenzo-thiazoline-6-sulfonic acid) substrate for peroxidase. The spectrophotometric absorbance of the samples was recorded using Microplate Reader (Bio-Rad Model 680, Hercules, CA) at 405 nm. Percent apoptosis was calculated using the formula: (100×treatment cell absorbance/control cell absorbance)−100.

### Statistical analysis

The statistical significance of differences between the values of SFN-treated and non-SFN- treated controls was determined by using Kruskal-Wallis with Dunn's post test using GraphPad Prism version 4.00 for Windows, GraphPad Software, San Diego, California, USA, www.graphpad.com. In each case, *P*<0.05 was considered statistically significant.

## Results

### SFN inhibits proliferation of human breast cancer cells at concentrations that have negligible effects on normal control breast cell

**Figure 1:**
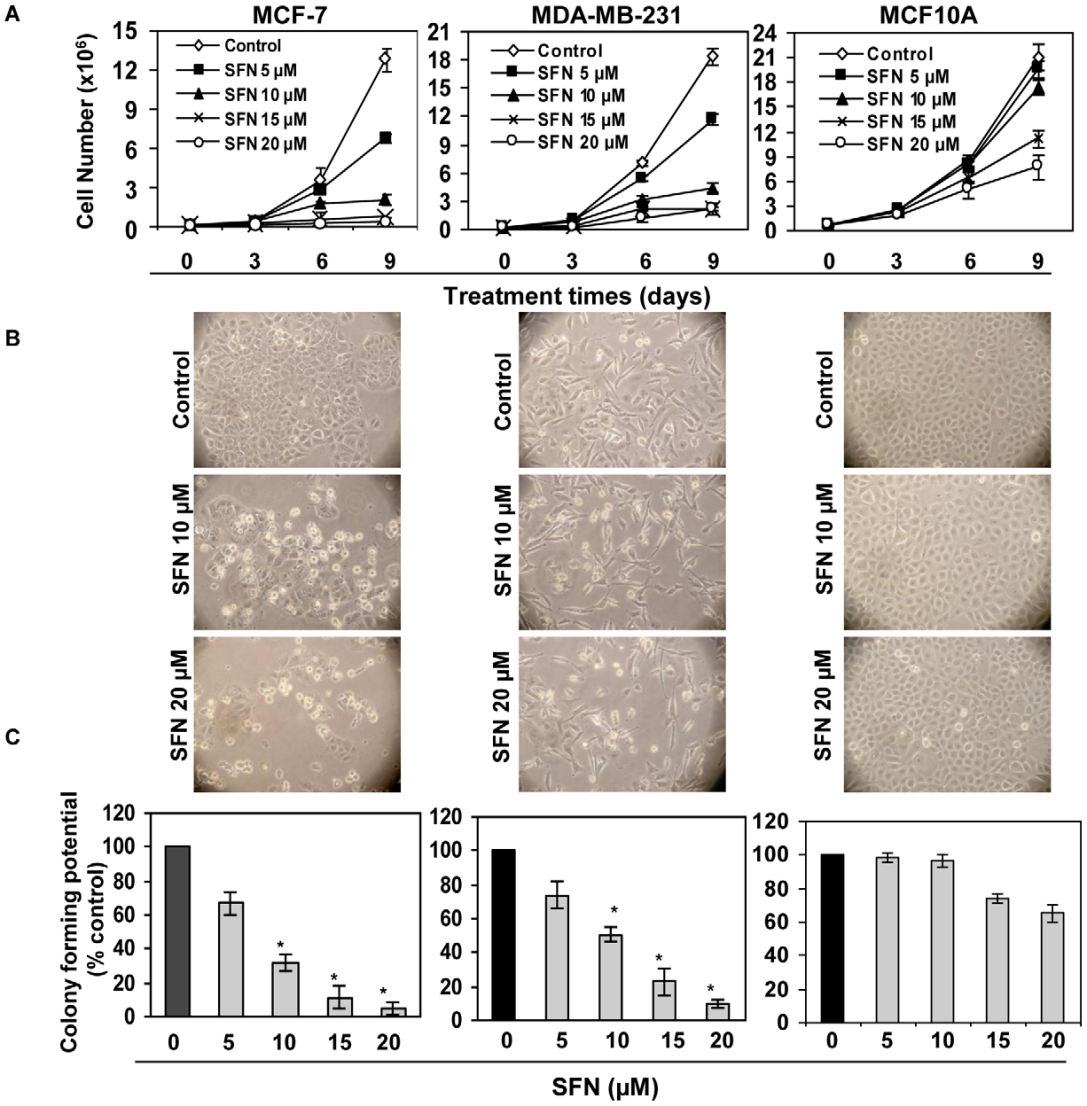
SFN inhibits proliferation of breast cancer cells but has negligible effect on control MCF10A cells up to 10 µM SFN. *A)* Breast cancer MCF-7 (left panel) and MDA-MB-231 (middle panel) cells as well as control MCF10A cells (right panel) were treated with SFN (0–20 µM) for 3, 6 and 9 days. Growth curve kinetics was obtained by counting the total number of viable cells at the indicated time intervals using trypan blue staining. Results were obtained from three independent experiments, mean ± SD. *B)* Morphological changes with SFN treatment on MCF-7 (left panel), MDA-MB-231 (middle panel) and MCF10A (right panel) cells. The white floating cells are indicative of apoptotic/dead cells. View, X100. *C)* Treatment with SFN (0–20 µM) inhibits the proliferation potential of human breast cancer MCF-7 (left panel) and MDA-MB-231 (middle panel) cells in a dose-dependent manner. Control MCF10A cells (right panel) did not show a significant inhibition of colony forming potential at lower doses of SFN. Proliferation of cells was assayed by the colonogenic assay. Colonies were stained using trypan blue and the total number counted at the end of the 15-day period protocol. The experiment was repeated three times and each point indicates the mean ± SD of the number of colonies formed. ^*^P<0.05.

To determine the effective dose of SFN on breast cancer cells, we first performed cell growth, morphological analysis and colonogenic assays to detect cell proliferation status. As shown in Fig. 1, human breast cancer MCF-7 (left panel) and MDA-MB-231 (middle panel) cells as well as normal control human breast MCF10A (right panel) cells were treated with the indicated concentrations of SFN for 3, 6 and 9 days for cell growth kinetics (Fig 1*A*) and colonogenic assays (Fig 1*C*). We observed a dose- and time-dependent cell growth inhibition with SFN treatment both in MCF-7 and MDA-MB-231 cells (Fig 1*A*). Doses of SFN up to 10 µM had negligible effects on cell growth and proliferation of the control MCF10A cells while these same doses inhibited these parameters for MCF-7 and MDA-MB-231 cells. In addition, cell growth was completely inhibited at 15 µM and 20 µM of SFN after 6 days of treatment. Control MCF10A cells were slightly inhibited in cell growth with 15 µM and 20 µM of SFN after 6 days of treatment, indicating that the 15 µM and higher SFN doses might be toxic to the normal breast cells. The morphology of human breast cancer cells treated with SFN was also changed as shown in Fig 1B. SFN-treatment clearly induced cell death and inhibited cellular proliferation in these breast cancer cells, whereas the equivalent SFN-doses were found to have very negligible cellular effects on normal MCF10A breast cells. We have also performed the colonogenic assay and found that treatment with SFN significantly reduces MCF-7 and MDA-MB-231 cell proliferation at doses of 10 µM SFN or higher, while very slight but non-significant cellular proliferation inhibition was found in control MCF10A cells (Fig 1*C*). These results indicate that SFN, at dosages of 10 µM or less, selectively inhibit breast cancer cells.

### SFN inhibits *hTERT* expression in breast cancer cells

**Figure 2:**
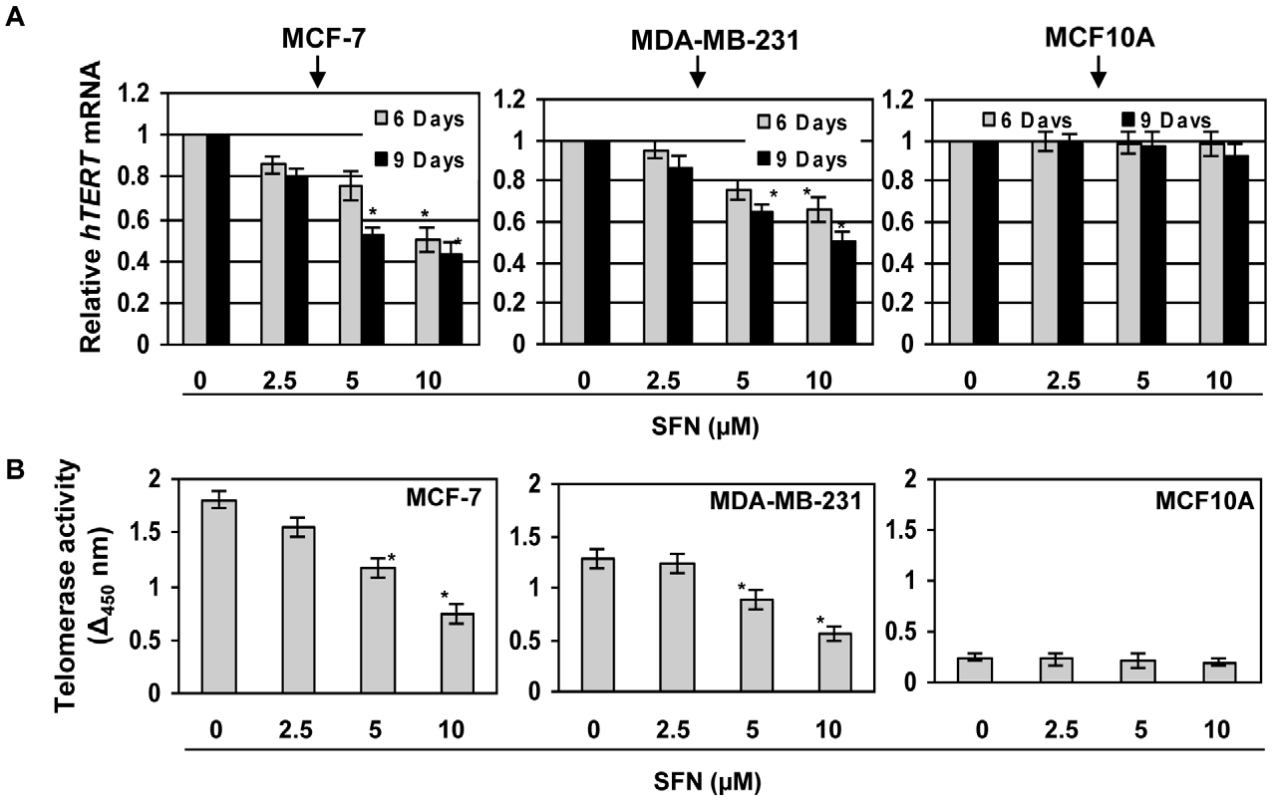
SFN inhibits telomerase in breast cancer cells. SFN inhibits *hTERT* mRNA expression in MCF-7 (left panel) and MDA-MB-231 (middle panel) human breast cancer cells but not in control MCF10A cells (right panel). *A)* Relative mRNA levels of *hTERT* in control (nontreated) as well as SFN (2.5–10 µM) treated cells were quantified at 6 and 9 days using real-time PCR. Data are in triplicates from three independent experiments and were normalized to *GAPDH*. The values were plotted against control as relative fold induction ± SD, ^*^P<0.05 is considered as significant. *B)* SFN inhibits telomerase activity in MCF-7 (left panel) and MDA-MB-231 (middle panel) human breast cancer cells but has negligible effect on control MCF10A cells (right panel). Telomerase activity was assayed with control (nontreated) as well as SFN-treated (2.5–10 µM) cells for 6 days. Telomerase activity was expressed as an arbitrary unit of absorbance at OD_450_–OD_690_. The experiment was repeated three times and each point indicates the mean absorbance ± SD, ^*^P<0.05.

It is well known that most of the cancer cells express elevated levels of telomerase, which allows these cells to survive, proliferate and bypass cellular senescence. Thus, it is important to assess the telomerase activity and *hTERT*, the key catalytic component of telomerase, alterations in these breast cancer cells with SFN-treatment. To investigate the effect of SFN on *hTERT* expression and telomerase activity, we performed real-time PCR and telomerase activity assay by ELISA, respectively. As shown in Fig. 2A, SFN at 5 µM or higher greatly inhibits *hTERT* expression in a dose- and time-dependent manner. The effect at 10 µM SFN is significant by 6 days in both MCF-7 (P<0.05) and MDA-MB-231 (P<0.05) cells. This is consistent with previous findings that inhibition of *hTERT* by chemopreventive compounds is one of the important contributing factors in cancer chemoprevention [31], [34]–[35]. We also assessed the effect of SFN on telomerase activity and observed a dose- and time-dependent inhibition in breast cancer cells. As illustrated in Fig. 2B, we found that SFN-treatment of breast cancer cells significantly reduced telomerase activity by 1.5- and 1.0-fold in MCF-7 (P<0.05) and MDA-MB-231 (P<0.05) cells, respectively. Control MCF10A cells had low level of telomerase activity (Fig. 2C) compared to breast cancer MCF-7 and MDA-MB-231 cells (Fig. 2A&2B). Treatment with SFN had very negligible telomerase inhibitory activity in normal MCF10A cells compared to untreated control cells. These results indicated that SFN acts on *hTERT* and leads to its down-regulation specifically on breast cancer cells, which may play a critical role in inhibition of cancer cell proliferation and survival.

### Alteration of methylation status leads to binding of CTCF to exon 1 of the *hTERT* gene

**Figure 3:**
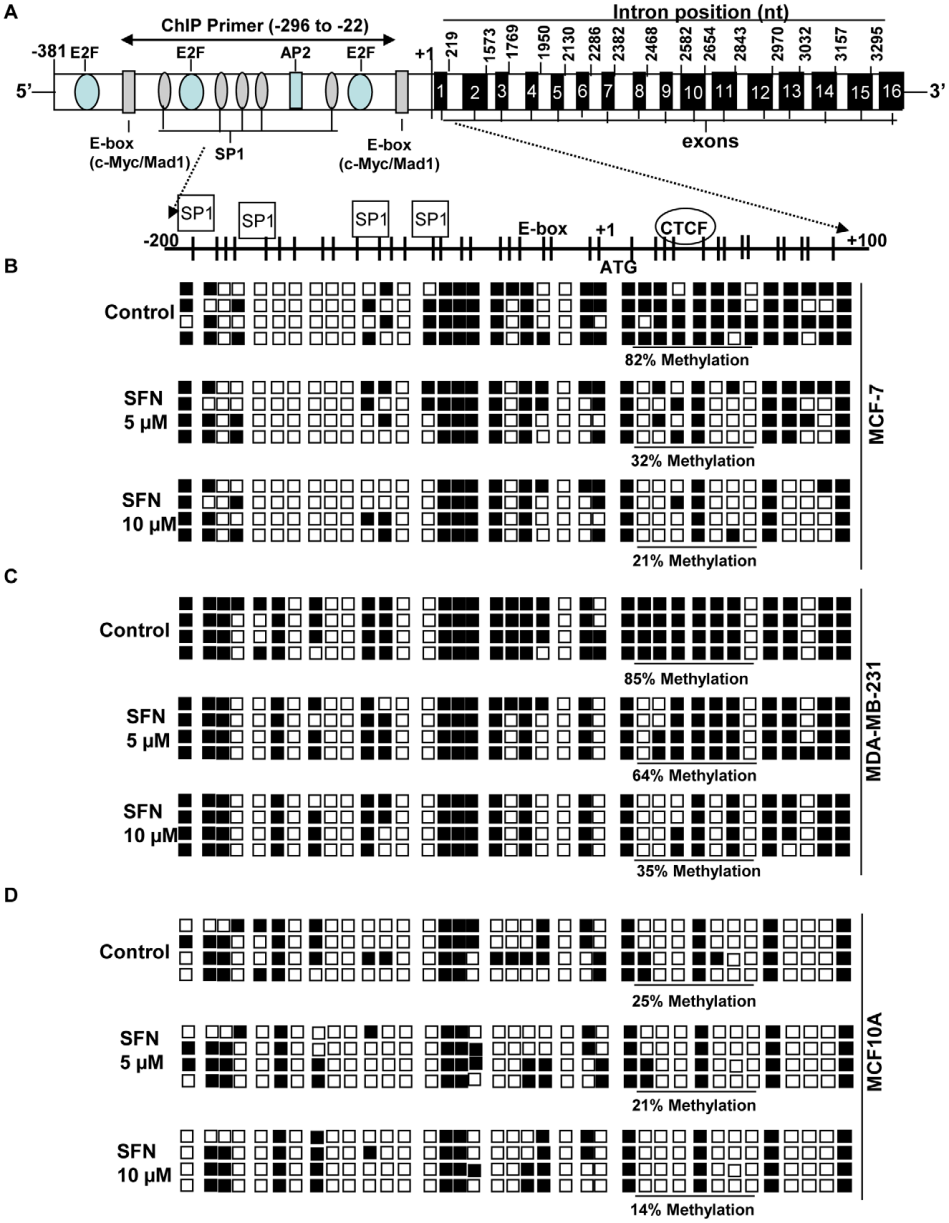
SFN induced methylation alteration of the *hTERT* promoter regions in normal and breast cancer cells. *A)* A generalized outline of the *hTERT* gene is shown with all 16 exons (15 introns) and other transcription factor binding sites. The *hTERT* promoter region contains distal E-box (binding site c-Myc/Mad1), −247 to −237, and the five SP1 sites, −187 to −179, −165 to −160, −133 to −125, −113 to −101, and −84 to −79. E2F binding sites are located at −313 to −308, −174 to −169 and −67 to −62. The proximal E-box is located at −34 to −29 from the translational starting site labeled as +1. The positions of exons 1 to 16 are shown in the *hTERT* gene and their intron nucleotide (nt) positions are shown at the end of the each exon. CpG density and various transcription factor binding sites in the *hTERT* promoter region are shown with the magnified dotted arrow. *B*) Methylation status of the *hTERT* promoter and 5′ exon region (−202 to +106 nucleotide) of breast cancer MCF-7 cells treated with SFN (0, 5, 10 µM) for 6 days. *C)* Methylation status of *hTERT* promoter and 5′ exon region (−202 to +106 nucleotide) of breast cancer MDA-MB-231 cells treated with SFN (0, 5, 10 µM) for 6 days. *D)* Methylation status of *hTERT* promoter and 5′ exon region (−202 to +106 nucleotide) of breast cancer MCF10A cells treated with SFN (0, 5, 10 µM) for 6 days. After PCR amplification of bisulfite-modified DNA and cloning into pGEM-T vector, several clones for each treatment were analyzed by DNA sequencing. Each square represents one CpG site. Filled squares: methylated; open squares: unmethylated. The number of methylated squares was counted from the total number of squares at the CTCF binding region for analyzing percent methylation at the CTCF binding region in the *hTERT* regulatory region.

**Figure 4:**
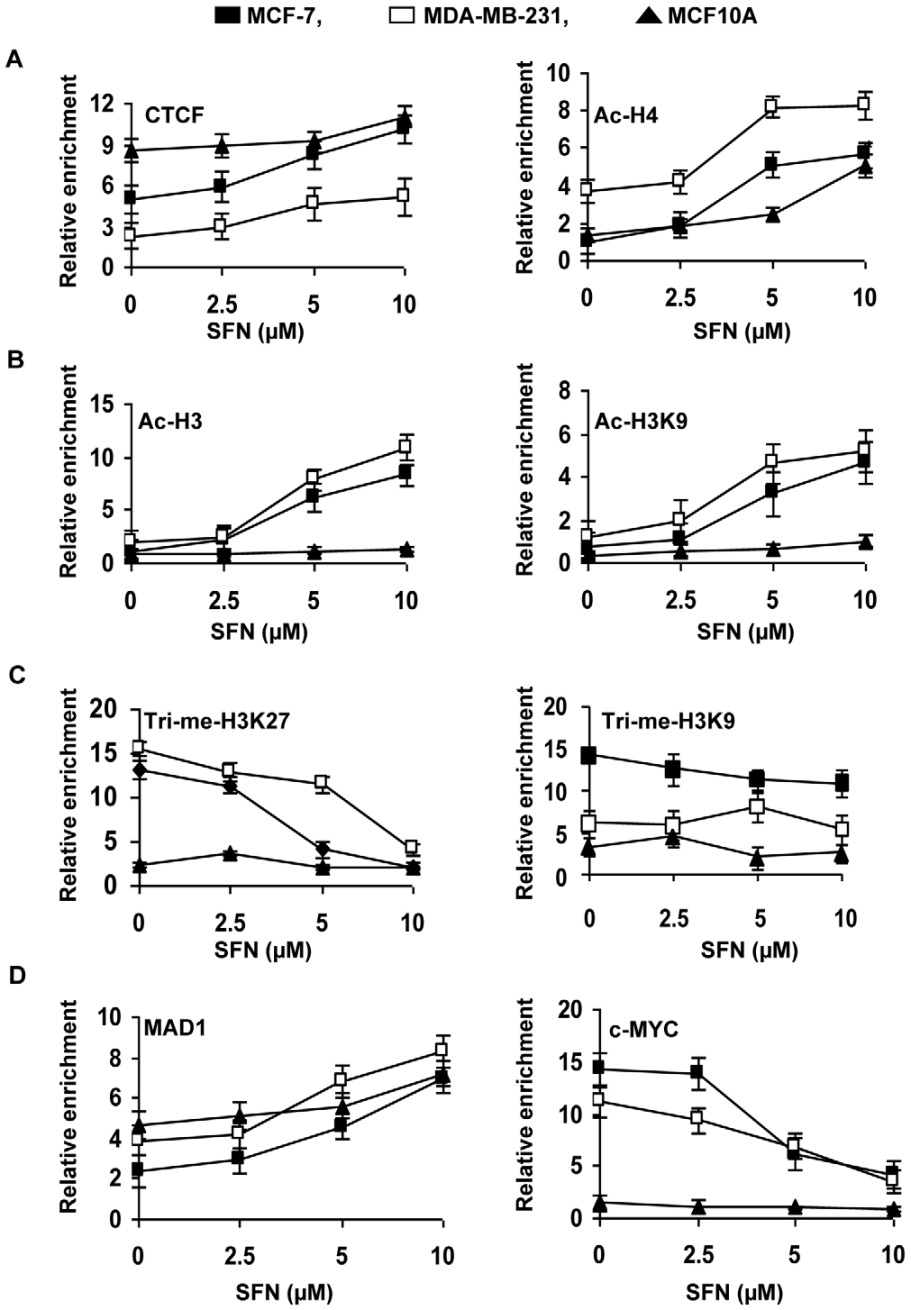
SFN induced histone modification changes of the *hTERT* promoter in normal and breast cancer cells. Breast cancer MCF-7 and MDA-MB-231 as well as non-tumorigenic MCF10A cells were treated with the indicated concentrations of SFN for 6 days, and analyzed by ChIP-qPCR assays using chromatin markers including acetyl-H3 (*B*, left panel) acetyl-H3K9 (*B*, right panel), acetyl-H4 (*A*, right panel), trimethyl-H3K27 (*C*, left panel) and trimethyl-H3K9 (*C*, right panel) as well as CTCF (*A*, left panel), MAD1 (*D*, left panel) and c-MYC (*D*, right panel) in the promoter region of *hTERT*. No antibody controls were also assessed to verify the ChIP efficiency. qPCR primers and conditions were used as described in *Materials and Methods*. The x axis represents the SFN doses inµM, and the y axis represents the relative enrichment of individual binding factors, the percentage of immunoprecipitates compared with the corresponding input samples (defined as 100). The experiment was repeated three times with triplicate in real-time PCR and each point indicates the mean ± SD.

It is well known that DNA methylation plays an important role in gene expression and regulation, especially *hTERT* expression [19]–[20], [31]. Further, to explore the molecular mechanism of SFN-induced repression of *hTERT* expression, we examined the methylation status of the *hTERT* regulatory region (from −202 to +106) in MCF-7 and MDA-MB-231 cells. A total of 37 CpG sites containing many overlapping transcription factor binding sites were analyzed for site-specific methylation status using bisulfite methylation sequencing analysis. However, there were only very slight methylation changes found in the core *hTERT* promoter region (−202 to −78) with SFN-treatment in both MCF-7 and MDA-MB-231 cells as well as control MCF10A cells (Fig 3*B, C, D*). In control (non-SFN-treated) MCF-7 and MDA-MB-231 cells, a very intense hypermethylation was found at the translation start site and CTCF binding region on the *hTERT* promoter, whereas, a very low level of methylation was observed in these regions in MCF10A cells. Conversely, the translation start site and CTCF binding site in the *hTERT* regulatory region was considerably demethylated with 5 µM SFN (50%) and 10 µM SFN (61%) treatment in MCF-7 cells (Fig 3*B*). Consistent with MCF-7 cells, MDA-MB-231 cells also underwent dramatic demethylation in these regions of the *hTERT* 5′-regulatory region with SFN-treatment in a dose-dependent manner. However, SFN treatment with MCF10A had a very negligible demethylation effect on the CTCF binding region of the *hTERT* regulatory region from 25% (SFN-untreated) to 14% (10 µM SFN-treated). It is known that CTCF binds to exon 1 of the *hTERT* gene and this methylation-sensitive transcription factor binding to *hTERT* drastically reduces *hTERT* expression [22]. CTCF binds to its unmethylated recognition sequence in the *hTERT* exon 1, whereas methylation of this site interferes with CTCF binding and reverses the gene expression [22]. Therefore, we asked whether SFN-mediated demethylation of the CTCF binding site enhances CTCF binding to the *hTERT* promoter in MCF-7 and MDA-MB-231 cells. Using quantitative-ChIP (q-ChIP) analyses, we found that treatment with SFN dose-dependently increases the CTCF binding to the *hTERT* exon 1 binding site both in MCF-7 and MDA-MB-231 (Fig 4*A*, left panel) cells. We also found elevated binding of CTCF to the *hTERT* promoter in SFN-untreated MCF10A cells and treatment with SFN slightly elevated the CTCF binding to the *hTERT* promoter. Therefore, SFN-mediated demethylation of CpGs of the CTCF binding sites may facilitate the binding of CTCF to the *hTERT* gene regulatory region to allow for CTCF-mediated down-regulation of *hTERT* expression in MCF-7 and MDA-MB-231 cells.

### Sulforaphane induced chromatin modification of the *hTERT* promoter

Previous studies have shown that *hTERT* is epigenetically regulated and its expression is often modulated by epigenetic processes [15]–[16], [35]. It is well established that SFN has HDAC inhibitory activity, which is one of the contributing factors for histone acetylation [9]–[10]. Since methylation changes were found at CpG sites of the CTCF region in the *hTERT* regulatory region, we sought to determine changes in histone modification of the *hTERT* regulatory region by SFN-treatment in MCF-7, MDA-MB-231 and non-tumorigenic MCF10A cells. SFN treatment resulted in enrichment of transcriptionally active chromatin markers, acetylated histone H3 (ac-H3) and H3 at lysine 9 (ac-H3K9) in both MCF-7 and MDA-MB-231 cells but not in MCF10A cells (Fig 4*B*). Acetylated histone H4 (ac-H4) were found to be elevated in all three breast cells, although MDA-MB-231 cells were found to have more enrichment of ac-H4 (fig 4*A*, right panel). We also found decreases in the methylation status of histone inactive markers such as trimethyl-H3 lysine 27 (tri-me-H3K27) and trimethyl-H3 lysine 9 (tri-me-H3K9) in MCF-7 and MDA-MB-231 cells with SFN-treatment (Fig 4*C*). SFN-treatment with MCF10A had negligible changes in tri-me-H3K9 and slight increases in tri-me-H3K27 levels. These changes of histone acetylation allow chromatin open structure to recruit repressor binding to the *hTERT* 5′-regulatory region [15], [19]. Furthermore, continuous SFN-treatment might also inhibit HDACs expression and their activity, due to the possible direct interaction of SFN with the HDACs active site, thereby inducing histone acetylation [36]. Active and inactive chromation modulations can control the antagonistic binding of MAD1 and c-MYC to the two E-boxes of the *hTERT* promoter, which are a major repressors and activators, respectively, of *hTERT* [16], [37]. Indeed, we found that the MAD1 repressor of *hTERT* is increased in its binding in response to SFN and the c-MYC activator is decreased in its binding to the *hTERT* promoter in MCF-7, MDA-MB-231 and MCF10A cells (Fig 4*D*). These results in combination with the results for CTCF binding (Fig 4*A*, left panel) provide key findings for the mechanisms of SFN-mediated *hTERT* inhibition in both MCF-7 and MDA-MB-231 breast cancer cells.

### Sulforaphane altered epigenetic enzymes expression and their activity

**Figure 5:**
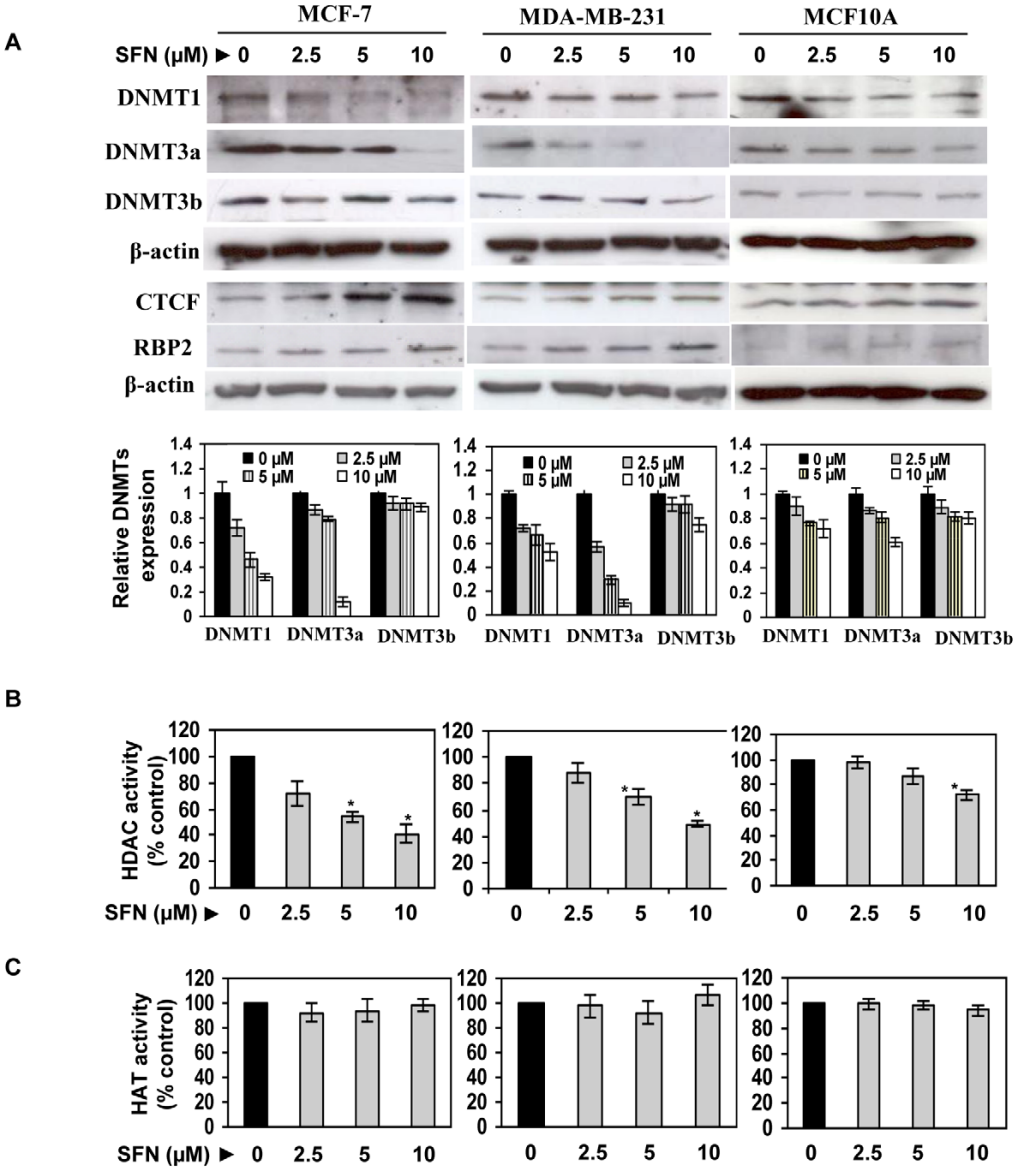
SFN altered epigenetic enzymes expression and their activity in normal and breast cancer cells. *A)* Effect of SFN on DNMTs, CTCF and RBP2 expression in human breast cancer MCF-7 (left panel) and MDA-MB-231 (middle panel) as well as non-tumorigenic MCF10A (right panel) cells. Cell lysates were prepared at 6 days after SFN-treatment at the indicated doses followed by western blotting to analyze DNMTs (DNMT1, DNMT3a and DNMT3b), CTCF and RBP2 expression. Actin was used as an equal loading control. Graphical representations are indicative of relative band intensity of DNMTs expression in MCF-7 (left panel), MDA-MB-231 (middle panel) and MCF10A (right panel) cells, normalized with β-actin. Values are mean of three independent experiments, band intensity ± SD. *B)* SFN inhibits HDAC activity. Breast cancer MCF-7 (left panel), MDA-MB-231 (middle panel) and control MCF10A (right panel) cells were treated with the indicated concentration of SFN for 6 days. Nuclear extracts were prepared and 20 µg of protein was used to estimate HDAC activity using the HDAC colorimetric assay kit. Values are representative of three independent experiments and represented as percent control ± SD; ^*^P<0.05. *C)* SFN treatment has no effect on the HAT activity. Breast cancer MCF-7 (left panel), MDA-MB-231 (middle panel) and control MCF10A (right panel) cells were treated with the indicated concentrations of SFN for 6 days. Nuclear extracts were prepared and 20 µg of protein was used to estimate HAT activity. Values are representative of three independent experiments and represented as percent control ± SD; ^*^P<0.05.

To further understand the epigenetic modulations that occurred in the *hTERT* 5′-control region, we assessed epigenetic-related enzymatic expression and activity of DNMTs (DNMT1, DNMT3a and DNMT3b), HDACs and HATs in MCF-7 (Fig 5, left panel), MDA-MB-231 (Fig 5, middle panel) and non-tumorigenic MCF10A cells (Fig 5, right panel), with SFN treatment. To our surprise, we discovered that SFN can considerably inhibit DNMT1 and 3a expression in a dose-dependent manner in human breast cancer cells and the inhibition was less in normal MCF10A cells. However, SFN has little if any effect on DNMT3b in MCF-7, MDA-MB-231 and MCF10A cells. As indicated in Fig 5A (graphical representation), 10 µM SFN in 6 days inhibited DNMT1 and DNMT3a expression in MCF-7 cells by 62% and 81%, respectively. In MDA-MB-231 cells, 10 µM SFN in 6 days inhibited DNMT1 and DNMT3a expression by 48% and 78%, respectively. The SFN-mediated inhibition of DNMTs expression could be an important contributing factor in facilitating demethylation at CTCF binding region on *hTERT* promoter observed in this study. Further, we also found that the *hTERT* repressor transcription factor, CTCF, is also increased in its expression with increasing RBP2 (a histone demethylase) expression in a dose-dependent manner with SFN treatment. It is known that SFN is an HDAC inhibitor; similarly, we have also found that SFN treatment significantly inhibited HDAC activity in these breast cancer cells. However, we did not find any considerable alterations in HAT activity with SFN treatment in these breast cells (Fig 5*C*). These results suggest that SFN-mediated HDAC activity allows chromatin remodeling for access of various transcription factors to the *hTERT* promoter; and DNMTs as well as RBP2-mediated demethylation facilitates repressors such as CTCF and MAD1 to bind to the *hTERT* gene control region, collectively contributing to *hTERT* repression in these breast cancer cells.

### Knockdown of CTCF restores *hTERT* expression and decreases apoptosis in SFN-treated breast cancer cells

**Figure 6:**
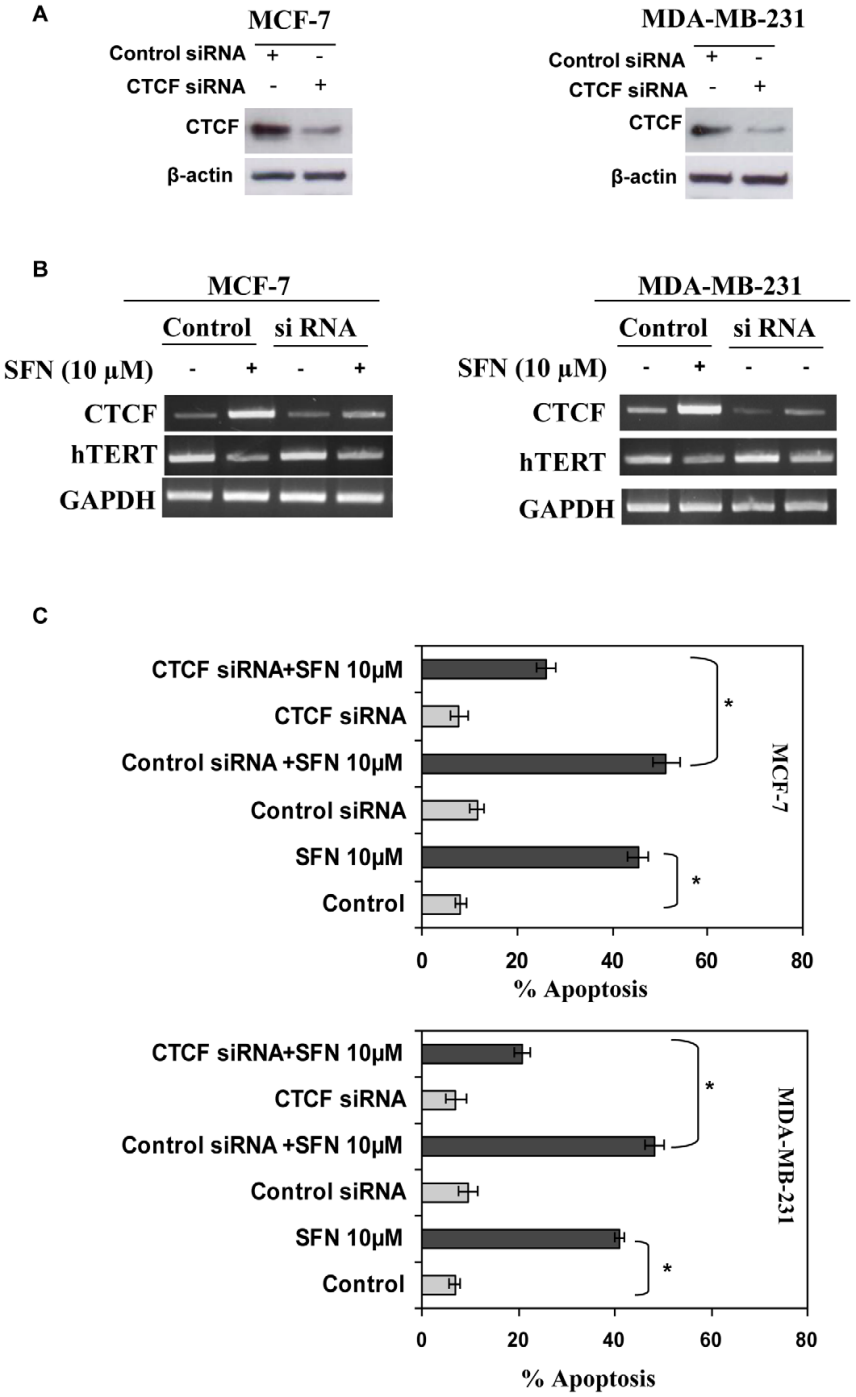
Knockdown of CTCF restores *hTERT* expression and decreases apoptosis in SFN-treated breast cancer cells. *A)* Breast cancer MCF-7 (left panel) and MDA-MB-231 (right panel) cells were subjected to treatments with 9 nM of CTCF siRNA or control siRNA fragments. Effects of siRNA interference with CTCF gene expression was assayed after 6 days using specific antibodies to CTCF and β-actin by western blot analysis. Data shown are representative of the three separate experiments. *B)* CTCF and control siRNA transfected cells were treated with 10 µM SFN for 6 days and analyzed for *CTCF* and *hTERT* mRNA expression by RT-PCR. *GAPDH* was used as an internal loading control. Photograph is representative of an experiment that was repeated in triplicate. *C)* CTCF and control siRNA-transfected, as well as non-transfected MCF-7 (top panel) and MDA-MB-231 (bottom panel) cells were treated with 10 µM SFN for 6 days. The cells were lysed with nuclear lysis buffer and assayed for apoptosis as described in *Materials and Methods*. Values are representative of three independent experiments. *P<0.05.

To further analyze the SFN-mediated repressive effect of *hTERT* expression by CTCF binding to the *hTERT* control region, we transiently transfected CTCF siRNA into the MCF-7 and MDA-MB-231 cells. Transfection of CTCF siRNA for 3 and 6 days considerably knocked down CTCF expression in both MCF-7 (Fig 6*A*, left panel) and MDA-MB-231 (Fig 6*A*, right panel) cells. In contrast with CTCF down-regulation, we found an elevated expression of *hTERT* mRNA in both human breast cancer cells in response to CTCF siRNA treatment (Fig 6*B*). We also found that partial knockdown of CTCF can partially reverse the inhibitory effect of SFN at 10 µM after 6 days of culture. The partial inhibition of *hTERT* expression with SFN-treated CTCF knockdown cells might be due to the binding of other transcription repressors such as MAD1, on the *hTERT* promoter (Fig 4*A*). However, from our results it is evident that knockdown of CTCF can reverse the inhibitory effect of SFN on *hTERT* expression. Further, we also analyzed the role of CTCF-regulated *hTERT* expression on SFN-induced apoptosis in breast cancer cells (Fig 6*C*). It was found that breast cancer cells treated with 10 µM SFN for 6 days significantly induced cellular apoptosis in both MCF-7 (P<0.05) and MDA-MB-231 cells (P<0.05). Conversely, SFN-induced cellular apoptosis was significantly reduced with CTCF knockdown with the restoration of *hTERT* expression in MCF-7 and MDA-MB-231 cells (Fig 6*B, C*). These results clearly indicate that CTCF is an important transcription factor required for SFN-mediated cellular apoptosis in human breast cancer cells. Therefore, SFN-induced cellular apoptosis is mediated, at least in part, by epigenetic modulation of CTCF binding to the *hTERT* regulatory exonic region and regulation of *hTERT* expression in human breast cancer cells is highly responsive to SFN treatments.

## Discussion

Botanical agents, particularly those that can be administered as dietary supplements, offer promising new options for the development of more effective chemopreventive and chemotherapeutic strategies. Sulforaphane (SFN) represents one such dietary botanical agent that has been indicated to have HDAC inhibitory activity [9]–[12]. Some HDAC inhibitors have been shown to have remarkable anti-tumor activity and are presently under clinical investigation [38]–[39]. Previous studies have demonstrated that exposure to HDAC inhibitors such as TSA can induce apoptosis and cell cycle arrest in various cancer cell lines [6], [26]–[27]. SFN has also been shown to have anti-proliferative and pro-apoptotic effects in many cancer cells, including breast cancer [3], [5]–[6]. In our present study, lower doses of SFN selectively inhibited cellular growth of breast cancer cells and had negligible effects on control breast cells. This is in accordance with previous findings that SFN induces cell type-specific apoptosis in breast cancer cells with activation of Bax/Bcl-2 and caspases [3]. However, the molecular triggers for induction or inhibitions of various genes specific to these pathways have not yet been fully elucidated. SFN-mediated HDAC inhibition activity causes a wide range of epigenetic alterations in many genes which are actively involved in malignant progression of cancer cells. HDAC inhibitors such as TSA induce histone hyperacetylation at the *hTERT* promoter and transactivate *hTERT* expression in telomerase-negative cells [24]. TSA-induced histone acetylation facilitates an open chromatin structure and allows for repressor protein binding to the *hTERT* promoter, which reduces *hTERT* transcription and leads to cellular apoptosis [19], [27].

In an attempt to identify potential epigenetic changes which mediate the effect of SFN on *hTERT* expression, we assessed the methylation status of CpG islands embedded in the *hTERT* control region (−202 to +106) in breast cancer as well as control non-tumorigenic MCF10A cells. In accordance with previous studies, the core promoter was partially methylated and an increased methylation pattern was identified at the first exonic region of the *hTERT* promoter in breast cancer cells [19]–[20]. Surprisingly, much less CpG methylation was found in the core promoter as well as the exon 1 region in MCF10A cells. SFN-induced dose-dependent demethylation of the exon 1 region which is located downstream of the transcriptional start site of the *hTERT* promoter might allow methylation-sensitive transcription factors such as CTCF to bind to the *hTERT* control region. CTCF is known to be an *hTERT* repressor and is associated with exon 1 of *hTERT* when the binding site is unmethylated [21]–[22]. Further, our ChIP analysis confirmed that SFN-induced demethylation at the first exon of the *hTERT* promoter results in increased binding of CTCF to the *hTERT* control region to allow for CTCF-mediated repression of *hTERT* transcription. Furthermore, unlike most human gene promoters in which CpG island demethylation leads to gene activation, *hTERT* control region demethylation is associated with transcriptional repression of *hTERT* expression [40].

Chromatin remodeling resulting from reversible acetylation of histones has been suggested to be a critical component of transcriptional regulation of *hTERT* expression [41]. In general, acetylation of histones leads to chromatin remodeling and facilitates transcriptional activation, whereas deacetylation causes transcriptional silencing. Histone acetylation and decetylation-modulated chromatin structure can be accessed with a number of transcription factors, including c-MYC and MAD1, which regulate gene expression by recruiting HATs and HDACs, respectively [41]. In accordance with earlier findings, we have also found that SFN treatment significantly inhibited HDAC activity in breast cancer cells; however, we did not find any significant alterations in HAT activity. In contrast, epigallocatechin-3-gallate (EGCG), one of the major constituents of green tea polyphenols, specifically inhibits HAT but not HDAC activity [42]. The SFN-mediated HDAC inhibition might be due to the possible direct interaction with SFN on the HDAC active site [36]. We found that SFN-induced chromatin alterations facilitate a dose-dependent enrichment of transcriptional active chromatin markers such as acetylated histone H3, H3K9 and acetyl-H4 in human breast cancer cells, whereas chromatin inactive markers such as trimethyl-H3K27 and trimethyl-H3K9 were decreased. Therefore, we provide several lines of evidence that SFN-mediated hyperacetylation facilitates the binding of various *hTERT* transcription repressors such as MAD1 and CTCF to the *hTERT* control region in breast cancer cells. Our results also suggest that SFN-induced MAD1 binding might recruit RBP2, a histone demethylase, which is responsible for the inhibition of chromatin inactive markers, thereby contributing to a stable repression of *hTERT* expression [23].

Another important discovery of this study is that SFN reduced DNMTs (DNMT1 and DNMT3a) activity in human breast cancer cells. DNMTs catalyze the methylation of genomic DNA. Of these, DNMT1 acts as a maintenance methyltransferase, whereas DNMT3a and DNMT3b exhibit *de novo* activity. In addition, DNMT1 induces hypermethylation of tumor suppressor genes to epigenetically repress their activation in tumorigenesis processes in many cancers including colon cancer [43]. Previously, we also have shown that genistein and EGCG result in down-regulation of the DNMTs which is directly associated with repression of *hTERT* expression through *hTERT* promoter demethylation in breast cancer cells [31], [44]. Numerous studies have also reported that DNA methylation plays important roles in *hTERT* transcriptional regulation [37], [44]. Together, our results suggest that SFN-induced down-regulation of DNMTs expression is not only involved in the demethylation processes of the *hTERT* control region in the process of anti-carcinogenesis, but also enhances binding of methylation-sensitive transcription factors such as CTCF to the *hTERT* regulatory region. Studies have shown that demethylating agents such as 5-azacytidine lead to a strong demethylation of the *hTERT* 5′-regulatory region, reactivation of CTCF binding and down-regulation of *hTERT* [21]. Convincingly, we found an inverse relationship between CTCF binding to the *hTERT* promoter with *hTERT* mRNA transcription in human breast cancer cells. In addition, CTCF siRNA experiments clearly demonstrated that depletion of CTCF restores the SFN-induced down-regulation of *hTERT* mRNA transcription in these breast cancer cells. Furthermore, down-regulation of *hTERT* expression facilitates the induction of cellular apoptosis in human breast cancer cells. This is consistent with previous findings that inhibition of *hTERT* by chemopreventive compounds is associated, at least in part, with the induction of cellular apoptosis [31], [34]–[35], [44]. Taken together, it is apparent that DNMTs-induced promoter demethylation and CTCF binding to the *hTERT* regulatory region are closely linked to the control of *hTERT* expression by SFN in breast cancer cells.

In the present study, we demonstrated not only SFN-induced down-regulation of telomerase in breast cancer cells but also explored possible epigenetic mechanisms such as demethylation at the first exon of *hTERT* and CTCF binding in relation to *hTERT* repression. It is important to point out that *hTERT* gene control is unique and the proposed mode of action is not the only way SFN inhibits cancer cell growth. The maximum concentrations used in this study were 10 µM and found to be the ideal dose for *in vivo* inhibition of HDAC activity in the colonic mucosa [38]. For humans to obtain concentrations of SFN similar to those we have used, one would have to consume about 1 cup (106 g) of broccoli sprouts per day based on *in vivo* studies which is well within practical limits [10], [38]. While this work is aimed at elucidating the mechanism by which SFN down-regulates *hTERT* expression, further *in vivo* confirmation is warranted. However, the SFN-induced epigenetic alterations observed in this and other investigations make it an attractive target for chemoprevention in varying cancer cell types.
